# Protective effects of *Quercus salicina* on alloxan-induced oxidative stress in HIT-T15 pancreatic β cells

**DOI:** 10.3892/etm.2013.885

**Published:** 2013-01-07

**Authors:** JIA-LE SONG, XIN ZHAO, QIANG WANG

**Affiliations:** 1 Department of Food Science and Nutrition, Pusan National University, Busan 609-735, Republic of Korea;; 2 Department of Biological and Chemical Engineering, Chongqing University of Education, Chongqing 400067, P.R. China

**Keywords:** *Quercus salicina*, pancreatic β cells, cell viability, reactive oxygen species, insulin secretion

## Abstract

The present study was designed to investigate the protective effect of hot water extracts from *Quercus salicina* leaves (QSWE) on alloxan-induced oxidative stress in HIT-T15 Syrian hamster pancreatic insulinoma cells. The HIT-T15 cells were treated with alloxan (1 mM) for 1 h and then co-incubated with the QSWE for 24 h. Alloxan significantly decreased the viability of the HIT-T15 cells (P<0.05). QSWE did not exhibit significantly cytotoxic effects and increased the viability of the HIT-T15 cells in a concentration-dependent manner. To further investigate the protective effects of QSWE on alloxan-induced oxidative stress in HIT-T15 cells, the cellular levels of reactive oxygen species (ROS), lipid peroxidation and endogenous antioxidant enzymes, including catalase (CAT), superoxide dismutase (SOD) and glutathione peroxidase (GSH-px), were analyzed. QSWE decreased the intracellular levels of ROS and lipid peroxidation and increased the activity of antioxidant enzymes. These results suggest that QSWE exerted cytoprotective activity against alloxan-induced oxidative stress in HIT-T15 cells through the inhibition of lipid peroxidation, reduction of ROS levels and stimulation of antioxidant enzyme activity. In addition, QSWE also increased the insulin secretion activity of the alloxan-treated HIT-T15 cells.

## Introduction

Diabetes mellitus has progressively become a serious public health problem worldwide. Reactive oxygen species (ROS)-induced pancreatic β cell death has an important role in the pathogenesis of diabetes and also affects insulin secretion. The ROS that are particularly responsible for oxidative stress include superoxide ions (O_2_^−^), hydroxyl radicals (^•^OH), singlet oxygen (^1^O_2_), hydrogen peroxide (H_2_O_2_), nitric oxide (NO) and peroxynitrite (ONOO^−^). Oxidative stress induces the dysfunction of pancreatic β cells, decreases insulin secretion (1) and leads to diabetic complications, including retinopathy, nephropathy, neuropathy and vascular damage (2,3). Generally, mammalian cells contain various antioxidative compounds, including low molecular mass antioxidants such as glutathione (GSH), uric acid, vitamin C and vitamin E, as well as various endogenous antioxidant enzymes against oxidative stress. Superoxide dismutase (SOD), catalase (CAT) and glutathione peroxidase (GSH-px) are three important endogenous antioxidant enzymes with roles against ROS-induced oxidative stress in living organs. Among these antioxidant enzymes, SOD catalyses the dismutation of the superoxide anion (O_2_^−^) into hydrogen peroxide (H_2_O_2_) which is transformed into H_2_O and O_2_ by CAT. GSH-px is key in removing lipid hydroperoxides and reducing free hydrogen peroxide to water.

Certain drugs which are used in clinical diabetes mellitus treatment are also associated with undesirable side-effects, such as gastrointestinal disturbances, edema, myocardial infarction and risk of cardiovascular disease (4,5). For these reasons, the development of more effective and safer drugs for treating diabetes has become essential. Currently, >400 traditional plant treatments for diabetes have been recorded (6). It is possible that anti-diabetic components from those natural plants may be ancillary medicines for diabetes.

*Quercus salicina* is an evergreen plant which grows in southern parts of the Korean Peninsula and Japan. It has exhibited anti-inflammatory, antiedemic, diuretic and litholytic activities and has been used to treat diarrhea, dysentery, dermatitis and hemorrhagia in Korean folk medicine (7–9).

The current study was designed to investigate the potential cytoprotective effects of QSWE on alloxan-induced oxidative stress and also to elucidate the mechanisms underlying its protective effects in HIT-T15 cells.

## Materials and methods

### Plant extract preparation

Fresh *Quercus salicina* leaves were purchased from a local market in Chongqing, China in August 2012. *Quercus salicina* hot water extract (QSWE) was prepared by boiling 100 g freeze-dried *Quercus salicina* leaves in 1 l distilled water for 2 h, followed by ultracentrifuging at 30,000 × g for 30 min, filtering with a 0.4-*μ*m filter, concentrating by heat evaporation and freeze-drying. The QSWE was redissolved in dimethyl sulfoxide (DMSO) at a concentration of 50 mg/ml and stored at 4°C until further study.

### Cell culture

HIT-T15 Syrian hamster insulin-secreting cells were obtained from the American Type Culture Collection (ATCC, Rockville, MD, USA). The cells were maintained in RPMI-1640 medium supplemented with 10% (v/v) fetal bovine serum (FBS) and 1% penicillin-streptomycin in a humidified CO_2_ incubator (Model 3154; Forma Scientific Inc., Marietta, OH, USA) with 5% CO_2_ at 37°C.

### Cell viability assay

Cell viability was assessed using 3-(4,5-dimethylthiazol-2-yl)-2,5-diphenyltetrazolium bromide (MTT) assays. Cells were seeded on 96-well plates at a density of 5×10^3^ cells/well. After a 24 h incubation, the cells were treated with alloxan (1 mM) for 1 h, then incubated with QSWE (2.5–50 *μ*g/ml) for 24 h. Following incubation, 100 *μ*l MTT reagent (0.5 mg/ml) was added to each well and the cells were incubated in a humidified incubator at 37°C to allow the MTT to be metabolized. After 4 h, the medium was removed and the cells were resuspended in formazan with 100 *μ*l DMSO. The absorbance of the samples was measured at 540 nm using a microplate reader (model 680; Bio-Rad, Hercules, CA, USA).

### Analysis of intracellular ROS

Intracellular ROS levels were measured using the fluorescent probe dihydrodichlorofluorescein diacetate (H2DCF-DA). Following treatment, the HIT-T15 cells were washed with calcium- and magnesium-free phosphate-buffered saline (PBS) and incubated in H_2_DCF-DA (20 *μ*M) containing serum- and phenol-red-free DMEM for 30 min. After incubation, the medium was removed and the cells were washed twice with PBS. Fluorescence was measured using a FLUOstar OPTIMA fluorescence plate reader (BMG Labtec, Ortenberg, Germany; excitation was read at 485 nm and emission at 535 nm). Relative ROS production (percentage of the control) was expressed as the ratio of the fluorescence of the treated samples to the response in the appropriate controls: (Fluorescence treatment / fluorescence control) × 100.

### Lipid peroxidation levels

Lipid peroxidation was evaluated by thiobarbituric acid (TBA)-reactive substance (TBARS) assays (10). In brief, the treated cells were washed with cooled PBS, scraped into trichloroacetic acid (TCA; 2.8%, w/v) and sonicated. Total protein was determined with a bicinchoninic acid (BCA) assay. The suspension was mixed with 1 ml TBA (0.67%, w/v) and 1 ml TCA (25%, w/v), heated (30 min, 95°C) and centrifuged (1,500 rpm, 10 min, 4°C). TBA reacts with the oxidative degradation products of lipids to yield red complexes that absorb at 535 nm. The amount of TBA-reactive substance was determined using a UV-2401PC spectrophotometer (Shimadzu, Kyoto, Japan).

### Antioxidant enzyme activity

HIT-T15 cells grown in 10-cm cell culture dishes were first treated with alloxan (1 mM) for 1 h and then incubated with QSWE (2.5–50 *μ*g/ml) for 24 h for further analysis. The cells were washed with PBS, detached by scraping and centrifuged, and the resulting cell pellet was stored at −80°C. Cell pellets were thawed, resuspended in 300 *μ*l cold lysis buffer (PBS, 1mM EDTA), homogenized and centrifuged (1,200 rpm, 10 min, 4°C). The resulting supernatants were used for activity measurements. CAT activity (U/mg protein) was measured according to the method described by Nelson and Kiesow (11) in which the disappearance of the substrate H_2_O_2_ was measured spectophotometrically at 240 nm. SOD activity (U/mg protein) was assayed using a modified autoxidation of pyrogallol method (12). One unit of SOD activity was defined as the amount of enzyme that inhibited the rate of autoxidation of pyrogallol by 50%. GSH-px activity (U/mg protein) was assayed according to the method of Hafemen *et al*(13). Protein contents were determined using a Bio-Rad protein assay kit according to the manufacturer’s instructions.

### Insulin secretion assay

Insulin secretion was measured with an ELISA assay. The cells were seeded at 5×10^5^ cells/well in 96-well plates. The cells were first treated with alloxan (1 mM) for 1 h and then treated with QSWE (2.5–50 *μ*g/ml) for 24 h. To measure the amount of insulin secreted, aliquots of samples (10 *μ*l/well) were collected from the experimental medium after the 24-h QSWE treatment and subjected to an insulin antiserum immunoassay according to the manufacturer’s instructions (LINCO Research, St. Charles, MO, USA). The absorbance was read at 450 and 590 nm in a microplate reader (model 680).

### Statistical analysis

Data were presented as the mean ± SD. Differences between the mean values for individual groups were assessed by one-way ANOVA with Duncan’s multiple range tests. P<0.05 was considered to indicate a statistically significant difference. The SAS v9.1 statistical software package (SAS Institute Inc., Cary, NC, USA) was used for the analyses.

## Results

### Effects of QSWE on alloxan-induced oxidative damage in HIT-T15 cells

**Figure 1.**
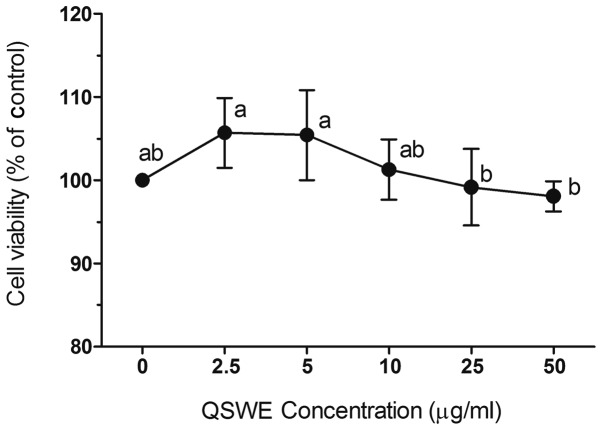
Effects of *Quercus salicina* leaf hot water extract (QSWE) on cell viability in HIT-T15 pancreatic cells. Data are representative of three independent experiments as mean ± SD. ^a–b^Mean values with different letters on the bars are significantly different from each other(P<0.05) according to Duncan’s multiple range test.

**Figure 2.**
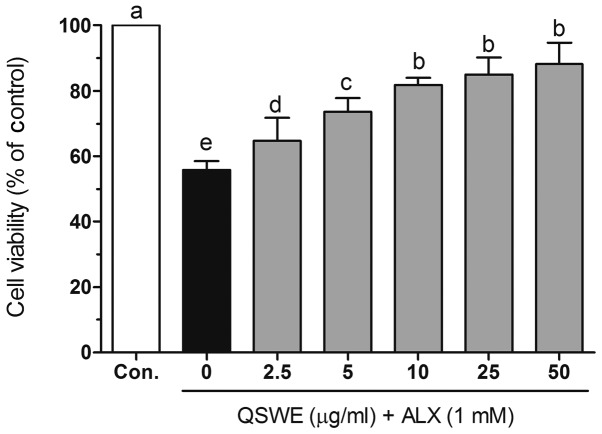
Effects of *Quercus salicina* leaf hot water extract (QSWE) on cell viability in 1 mM alloxan (ALX)-treated HIT-T15 pancreatic cells. Data are representative of three independent experiments as mean ± SD. ^a–e^Mean values with different letters on the bars are significantly different from each other (P<0.05) according to Duncan’s multiple range test.

To investigate QSWE-induced cytotoxicity, HIT-T15 cells were first treated with various concentrations of QSWE (2.5–50 *μ*g/ml) for 24 h and the cell viability was determined using MTT assays. QSWE did not exhibit any significant cytotoxicity and the cell viabilities were >90% (Fig. 1). Based on these results, concentrations between 2.5 and 50 *μ*g/ml were used for further studies. As shown in Fig. 2, alloxan (1 mM) significantly induced cell death in the HIT-T15 cells. However, following treatment with various concentrations of QSWE, the cell viability was increased in a concentration-dependent manner.

### Effects of QSWE on alloxan-induced intracellular ROS levels in HIT-T15 cells

**Figure 3.**
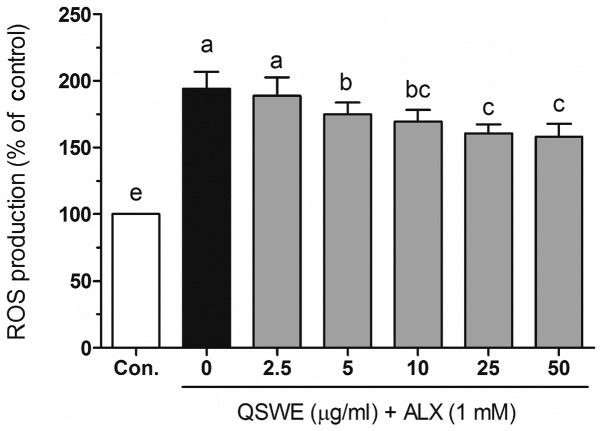
Effects of *Quercus salicina* leaf hot water extract (QSWE) on intra-cellular reactive oxygen species (ROS) levels in 1 mM alloxan (ALX)-treated HIT-T15 pancreatic cells. Data are representative of three independent experiments as mean ± SD. ^a–e^Mean values with different letters on the bars are significantly different from each other(P<0.05) according to Duncan’s multiple range test.

To investigate the protective effects of QSWE in alloxan-treated HIT-T15 cells, the intracellular ROS levels were evaluated using a fluorescent probe, H_2_DCF-DA. As shown in Fig. 3, alloxan significantly increased the ROS levels compared with those in the normal cells. In the presence of alloxan, QSWE significantly reduced ROS generation in a concentration-dependent manner between 2.5 and 50 *μ*g/ml. The intracellular ROS levels were 188.6±13.7, 174.9±8.9, 169.5±8.8, 160.5±6.8 and 158.1±9.8% at 2.5, 5, 10, 25 and 50 *μ*g/ml QSWE, respectively. Treatment with the same concentrations of QSWE alone did not significantly increase the intracellular ROS levels (data not shown). These results suggest that QSWE is a free radical scavenger.

### Effects of QSWE on lipid peroxidation in alloxan-treated HIT-T15 cells

**Figure 4.**
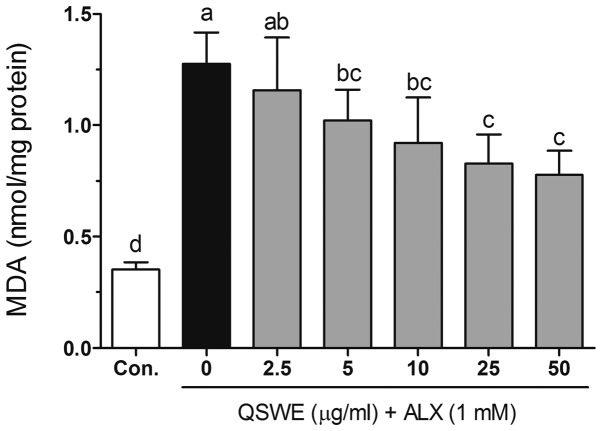
Effects of *Quercus salicina* leaf hot water extract (QSWE) on intracellular malonaldehyde (MDA) levels in 1 mM alloxan (ALX)-treated HIT-T15 pancreatic cells. Data are representative of three independent experiments as mean ± SD. ^a–d^Mean values with different letters on the bars are significantly different from each other (P<0.05) according to Duncan’s multiple range test.

Free radicals and ROS-induced oxidative damage were markedly associated with the lipid peroxidation of cell membranes and increased the levels of malondialdehyde (MDA), which is a biomarker of cell membrane lipid peroxidation. As shown in Fig. 4, alloxan significantly increased the level of MDA (to 1.27±0.14 nmol/mg protein) compared with that in the normal cells (0.35±0.03 nmol/mg protein). QSWE significantly reduced the MDA levels in a concentration-dependent manner between 2.5 and 50 *μ*g/ml. The MDA levels were 1.16±0.23, 1.02±0.14, 0.92±0.20, 0.83±0.13 and 0.78±0.11 nmol/mg protein at 2.5, 5, 10, 25 and 50 *μ*g/ml QSWE, respectively.

### Effects of QSWE on the activity of antioxidant enzymes in alloxan-treated HIT-T15 cells

Table I shows the intracellular antioxidant enzyme activities of QSWE in the alloxan-treated HIT-T15 cells. The activity of SOD was reduced by alloxan (to 7.25±0.68 U/mg protein) and this reduction was attenuated by various concentrations of QSWE; the SOD activity was 7.76±1.07, 8.85±1.26, 10.37±0.57, 10.65±1.65 and 11.60±1.18 U/mg protein at 2.5, 5, 10, 25 and 50 *μ*g/ml QSWE, respectively. Following treatment with alloxan, the cellular CAT activity was reduced (1.25±0.15 U/mg protein) compared with that in the normal cells (2.11±0.24 U/mg protein). However, the reduction in CAT activity was significantly attenuated (P<0.05) by treatment with QSWE. In addition, QSWE also attenuated the alloxan-induced reduction in GSH-px activity in the HIT-T15 cells. The GSH-px activity of the alloxan-treated cells significantly increased following treatment with QSWE; the increased levels ranged from 3.29±0.15 to 4.85±0.20 U/mg protein.

### Effects of QSWE on insulin secretion in alloxan-treated HIT-T15 cells

**Figure 5.**
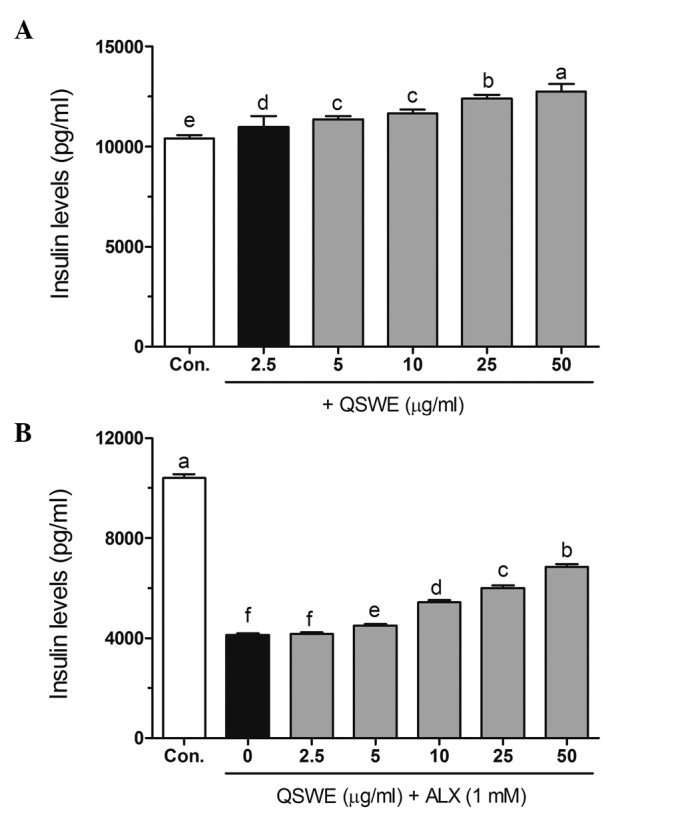
Effects of *Quercus salicina* leaf hot water extract (QSWE) on insulin levels in (A) normal and (B) 1 mM alloxan (ALX)-treated HIT-T15 pancreatic cells. Data are representative of three independent experiments as mean ± SD. ^a–f^Mean values with different letters on the bars are significantly different (P<0.05) according to Duncan’s multiple range test.

As shown in Fig. 5A, QSWE effectively increased insulin secretion in normal HIT-T15 cells. However, alloxan significantly decreased the insulin level (4,119.58±66.70 pg/ml) compared with that in the normal cells (10,411.66±159.14 pg/ml). Following treatment with QSWE, the insulin levels in the alloxan-treated cells were 4,160.78±67.36, 4,490.35±72.70, 5,437.85±88.04, 6,014.59±97.38 and 6,846.02±116.42 pg/ml at at 2.5, 5, 10, 25 and 50 *μ*g/ml QSWE, respectively (Fig. 5B). These results suggest that QSWE treatment is effective for increasing pancreatic β cell survival and maintaining normal biological function in ROS-induced diabetes.

## Discussion

ROS-induced oxidative damage in pancreatic β cells is considered to have an important role in the pathological process of diabetes. Certain studies have reported that reducing ROS levels and treatment with antioxidants (such as NAC, vitamin C and vitamin E) are able to improve β cell structure and function *in vitro*(14,15). However, whether QSWE protects pancreatic β cells from alloxan-induced oxidative damage has not been investigated. The present study demonstrated that QSWE was able to protect HIT-T15 cells from ROS-induced cell damage. The cytoprotective effects are mainly mediated by upregulated intracellular antioxidant enzyme activity.

In the present study, it was revealed that QSWE prevented alloxan-induced cell death, as assessed by MTT assays. The results showed that QSWE alone was not significantly cytotoxic to cells at the tested concentrations. Treatment with QSWE exhibited significant protective effects which may be due to the free radical scavenging activity of QSWE.

To evaluate the role of the free radicals in the protective activity of QSWE, the effect on alloxan-induced ROS generation was analyzed using H_2_DCF-DA assays. Treatment with alloxan alone significantly increased the intracellular ROS generation. Following treatment with QSWE, ROS generation was observed to decline in a concentration-dependent manner. This decrease in alloxan-induced ROS may account for the observed cytoprotective effect.

Lipid peroxidation is the most extensively investigated process induced by free radicals. ROS participate in the toxic actions that lead to the apoptosis of insulin-producing cells. In the present study, increased lipid peroxidation levels were observed in alloxan-treated HIT-T15 cells. However, treatment with QSWE resulted in a decrease in lipid peroxidation, indicating that oxidative stress-related damage was lower in the QSWE-treated cells. The capacity of QSWE to reduce lipid peroxidation may be due to its function as a preventive antioxidant for scavenging initiating radicals.

Overproduced free radicals are scavenged by endogenous antioxidant enzymes, including SOD, CAT and GSH-px. In cells, SOD catalyzes the conversion of superoxide (O_2_^−^) to hydrogen peroxide (H_2_O_2_) and H_2_O_2_ is further reduced H2O by the activity of CAT or GSH-px. Pancreatic β cells have been reported to contain low levels of endogenous antioxidant enzymes, in particular GSH-px and CAT (16). In the present study, alloxan significantly decreased the activity of GSH-px and CAT in HIT-T15 cells. However, QSWE treatment increased the activity of these antioxidant enzymes in the alloxan-treated HIT-T15 cells, indicating that QSWE was able to reduce alloxan-induced oxidative stress. Certain studies have reported that the overexpression of Cu/Zn-SOD showed a protective effect against nitric oxide-induced cytotoxicity in human islets and INS-1 insulin-secreting cells (17) and alloxan- and streptozotocin-induced diabetes (18,19). CAT also showed a protective effect against H_2_O_2^−^_ and streptozotocin-induced oxidative stress *in vivo*(20). In addition, the combined over-expression of CAT and GSH-px also revealed a protective effect against ROS-induced oxidative stress by increasing the activity of Cu/Zn SOD or MnSOD (21–23).

In conclusion, in the present study, QSWE demonstrated protective activity against alloxan-induced cell death in HIT-T15 hamster insulin-secreting cells. QSWE was able to effectively scavenge alloxan-induced intracellular ROS and prevent pancreatic β cell death by increasing the activity of the intracellular antioxidant enzymes SOD, CAT and GSH-Px. Furthermore, QSWE also promoted insulin secretion in the alloxan-treated HIT-T15 cells.

## Figures and Tables

**Table I.** Effect of QSWE on the activity of CAT, SOD and GSH-px in HIT-T15 cells exposed to alloxan.

Group	QSWE concentration (*μ*g/ml)	CAT (U/mg protein)	SOD (U/mg protein) GSH-	px (U/mg protein)
Normal	-	2.11±0.24^a^	14.78±0.40^a^	5.34±0.35^a^
ALX (1 mM) + QSWE	0.0	1.25±0.15^c^	7.25±0.68^d^	3.19±0.24^e^
	2.5	1.68±0.24^b^	7.76±1.07^d^	3.29±0.15^e^
	5.0	1.70±0.14^b^	8.85±1.26^cd^	3.74±0.32^d^
	10.0	1.89±0.23^ab^	10.37±0.57^bc^	4.19±0.16^c^
	25.0	1.95±0.07^ab^	10.65±1.65^b^	4.43±0.18^c^
	50.0	2.04±0.14^a^	11.60±1.18^b^	4.85±0.20^b^

Data are representative of three independent experiments as mean ± SD.

a–e Mean values with different letters are significantly different from each other (P<0.05) according to Duncan’s multiple range test. QSWE, *Quercus salicina* leaf hot water extract; CAT, catalase; SOD, superoxide dismutase; GSH-px, glutathione peroxidase; ALX, alloxan.
